# A Flexible Tribotronic Artificial Synapse with Bioinspired Neurosensory Behavior

**DOI:** 10.1007/s40820-022-00989-0

**Published:** 2022-12-29

**Authors:** Jianhua Zeng, Junqing Zhao, Tianzhao Bu, Guoxu Liu, Youchao Qi, Han Zhou, Sicheng Dong, Chi Zhang

**Affiliations:** 1 CAS Center for Excellence in Nanoscience, Beijing Key Laboratory of Micro-Nano Energy and Sensor, Beijing Institute of Nanoenergy and Nanosystems, Chinese Academy of Sciences, Beijing, 101400 People’s Republic of China; 2 Center on Nanoenergy Research, School of Physical Science and Technology, Guangxi University, Nanning, 530004 People’s Republic of China; 3 School of Nanoscience and Technology, University of Chinese Academy of Sciences, Beijing, 100049 People’s Republic of China

**Keywords:** Flexible electronics, Tribotronics, Artificial synapses, Contact electrification, Neurosensory behavior

## Abstract

As key components of artificial afferent nervous systems, synaptic devices can mimic the physiological synaptic behaviors, which have attracted extensive attentions. Here, a flexible tribotronic artificial synapse (TAS) with bioinspired neurosensory behavior is developed. The triboelectric potential generated by the external contact electrification is used as the ion-gel-gate voltage of the organic thin film transistor, which can tune the carriers transport through the migration/accumulation of ions. The TAS successfully demonstrates a series of synaptic behaviors by external stimuli, such as excitatory postsynaptic current, paired-pulse facilitation, and the hierarchical memory process from sensory memory to short-term memory and long-term memory. Moreover, the synaptic behaviors remained stable under the strain condition with a bending radius of 20 mm, and the TAS still exhibits excellent durability after 1000 bending cycles. Finally, Pavlovian conditioning has been successfully mimicked by applying force and vibration as food and bell, respectively. This work demonstrates a bioinspired flexible artificial synapse that will help to facilitate the development of artificial afferent nervous systems, which is great significance to the practical application of artificial limbs, robotics, and bionics in future.

**Supplementary Information:**

The online version contains supplementary material available at 10.1007/s40820-022-00989-0.

## Highlights

- A flexible tribotronic artificial synapse with bioinspired neurosensory behaviour was demonstrated, which establishes an active interaction mechanism with the environment.
- The device can well exhibit tuneable synaptic behaviours by changing the mechanical input modes, including excitatory postsynaptic current, paired-pulse facilitation, and the hierarchical memorial process.
- The device has excellent mechanical flexibility that can exhibit stable synaptic functions even under strain conditions with a bending radius of 20 mm after 1000 bending cycles.

**Supplementary Information:**

The online version contains supplementary material available at 10.1007/s40820-022-00989-0.

## Graphical abstract

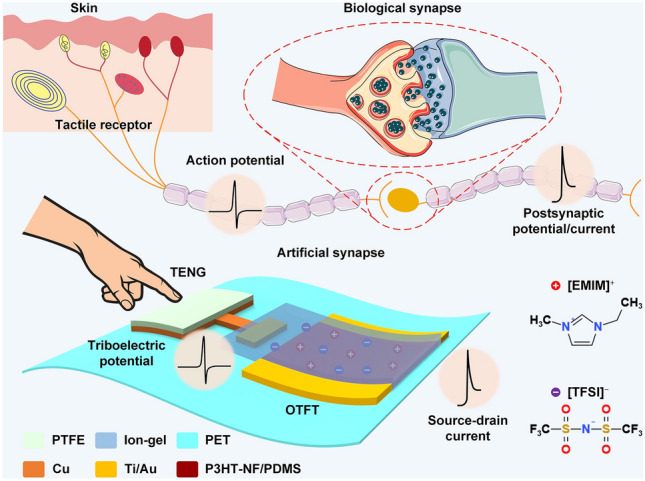

## Introduction

In the biological afferent nervous system, the perception, transmission and processing of external stimulus information depend on the distributed and parallel networks of receptors, synapses and neurons, which have significant advantages in dealing with unstructured and complex real-world problems [1–8]. Biological synapse is the basic unit of the nervous system, and synaptic plasticity is considered to underlie cognition, learning and memory for various complex information [9–12]. Therefore, it is of great significance for the intelligent development of artificial limbs, robotics, and bionics to endow electronic devices with the ability of synaptic-like behaviors [13–16]. In recent years, many types of synaptic devices have been developed based on the field effect transistor, which can mimic synaptic behaviors by using the electrical output characteristics [17–21]. However, these synaptic devices are usually gated by external voltage source, lacking active interaction mechanism with the external environment. It is thus necessary to develop synaptic devices with direct interaction behavior to build artificial neural afferent systems.

Since invented in 2012, triboelectric nanogenerator (TENG) has been proved to be an effective energy conversion technology that can convert mechanical energy in the environment into electrical energy [22–25], which has been widely applied in the fields of micro/nano power sources [26, 27], self-powered sensing [28, 29], blue energy [30, 31], and high voltage sources [32, 33]. Specifically, the coupling between the triboelectric potential generated by TENG and semiconductor devices enables tuning of the carrier transport, thereby establishing an active interaction mechanism between the environment and electronics, thus initiating an emerging field of tribotronics [34–36]. The semiconductor devices based on tribotronics have been successfully verified in logic control [37], touch switch [38], gas monitoring [39] and tactile sensing [40]. Ulteriorly, the coupling of the triboelectric potential and synaptic transistor devices has also successfully realized the active interaction mechanism between artificial synapses and mechanical stimuli, but most of these artificial synapses use silicon as the substrate, resulting in their lack of biocompatibility, which greatly limits their application in bionics [41].

Herein, we reported a flexible tribotronic artificial synapse (TAS) with bioinspired neurosensory behavior. The triboelectric potential generated by the external contact electrification is used as the ion-gel-gate voltage of the organic thin film transistor (OTFT), which can tune the carriers transport through the migration/accumulation of ions. The TAS successfully demonstrates a series of synaptic behaviors by external stimuli, such as excitatory postsynaptic current (EPSC), paired-pulse facilitation (PPF), and the hierarchical memory process from sensory memory (SM) to short-term memory (STM) and long-term memory (LTM). Moreover, the synaptic behaviors remained stable under the strain condition with a bending radius of 20 mm, and the TAS still exhibits excellent durability after 1000 bending cycles. Finally, the Pavlovian conditioning has been successfully mimicked by applying force and vibration as food and bell, respectively. This work demonstrates a bioinspired flexible artificial synapse that will help to facilitate the development of artificial afferent nervous systems, which is great significance to the practical application of artificial limbs, robotics, and bionics in future.

## Experimental Section

### Materials

Poly(3-hexylthiophene-2,5diyl) (P3HT), 1-ethyl-3-methylimidazolium bis(trifluoromethylsulfonyl)imide (EMIM-TFSI), and poly(vinylidene fluoride-co-hexafluoropropylene) (PVDF-HFP) were all purchased from Sigma-Aldrich. Anhydrous m-xylene (> 99%) was purchased from Aladdin. Poly(dimethylsiloxane) (PDMS) was purchased from Dow Corning. Acetone (> 99.5%) purchased from Xilong Science Co., Ltd.

### Preparation Process

#### Preparation of P3HT-NF/PDMS Semiconductor Solution

First, P3HT was completely dissolved in *m*-xylene solvent at 60 °C, and then naturally cooled to room temperature (Fig. S2a, bright orange). PDMS (Dow Corning Sylgard 184, crosslinker:prepolymer = 1:10 (w/w)) was completely dissolved in *m*-xylene solvent. Then the two solutions were mixed. To promote the formation of crystalline P3HT, the mixed solution was aged at room temperature for 1 h. Finally, the mixed solution was cooled at −15 °C for 30 min, and then naturally rose back to room temperature (Fig. S2b, dark purple).

#### Preparation of Ion-Gel Dielectric Film

The ion-gel dielectric solution was composed of the PVDF-HFP, ionic liquid EMIM-TFSI and acetone in a weight ratio of 1:4:7 and completely dissolved at 70 °C. The ion-gel dielectric film was prepared by casting the solution on a clean silicon wafer and cured in a vacuum oven for 24 h to remove residual solvents.

#### Preparation of Device

First, the Ti/Au electrodes of the OTFT were prepared through a shadow mask by means of magnetron sputtering on a thin flexible PET film. Secondly, the P3HT-NF/PDMS semiconductor solution was spin coated on the channel area of the source-drain electrodes at 2000 rpm for 60 s through a Kapton shadow mask, and annealed at 90 °C for 30 min. Then, the ion-gel dielectric film was aligned and heat laminated on the device using a self-made clamp. Finally, the Cu and PTFE films were assembled on the PET film in sequence, in which the Cu film was connected to the OTFT as the gate electrode.

### Characterization

The impedance analysis of ion-gel dielectric was carried out using Keysight E4990A impedance analyzer. The surface morphology and thickness of P3HT-NF/PDMS film was characterized by atomic force microscope (AFM, Veeco Dimension 3000). The optical microscope image of the OTFT was by optical microscope (Zeiss, Axioscope AI). The electrical out characterizations of the TAS were measured by using a Keithley 2612B source meter. The contact-separation process of the TENGs was controlled by a linear motor and a vibration shaker, respectively.

## Results and Discussion

### Design of the TAS

**Fig. 1:**
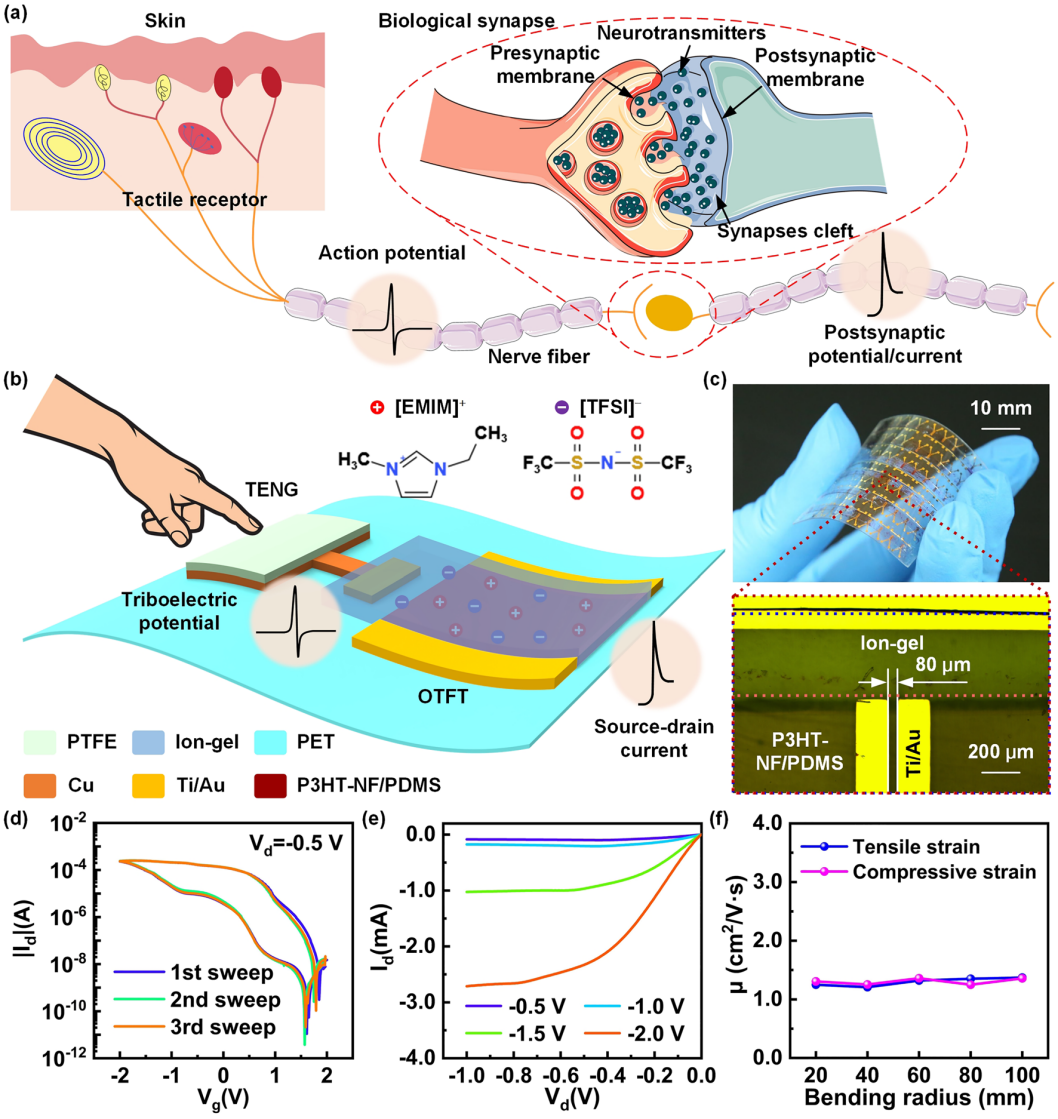
Biological afferent nervous system and TAS device. **a** Schematic diagram of the biological afferent nervous system. **b** Schematic illustration of the TAS device. **c** Optical microscope image of the OTFT. **d** Double-sweep transfer characteristics of the OTFT. **e** Output characteristics of the OTFT. **f** The mobility of the OTFT under different bending radius in tensile and compressive strain states

Figure 1A shows the biological afferent nervous system, which consists of tactile receptors, biological synapses, and neuron fibers. The tactile receptors can convert stimuli signals received by the skin into electrical spikes, which are subsequently transmitted to biological synapses with processing function through nerve fibers. The electrical spikes can be transmitted to the presynaptic membrane as action potential, so that abundant neurotransmitters are released to synapses cleft, and then act on the postsynaptic membrane to induce postsynaptic potential/current [42]. The signal processing of biological synapses depends on the transmission of neurotransmitters in synapses cleft. To mimic the neurosensory behavior, we designed a flexible TAS as showed in Fig. 1b. The TAS is fabricated on a thin flexible PET film to meet the mechanical compatibility requirements of curved and dynamic surfaces. First, the Ti/Au electrodes of OTFT are sputtered on the PET film. Then, the P3HT-NF/PDMS semiconductor solution is spin coated on the channel area of the source-drain electrodes. Subsequently, the ion-gel dielectric film is aligned and heat laminated on the device. Finally, the Cu and PTFE films were assembled on the PET film in sequence, in which the Cu film was connected to the OTFT as the gate electrode. In this flexible TAS, the triboelectric potential generated by the external contact electrification is regarded as the action potential, and the source-drain current (*I*_*d*_) of the OTFT is analogous to the postsynaptic current. Figure 1c displays the OTFT device in the bending state, and the optical microscope image of the magnified OTFT in top view. The channel length of the OTFT device is 80 μm.

Specifically, ion-gel with high capacitance and P3HT polymer semiconductor with high mobility are used as the gate dielectric layer and the semiconductor layer of the OTFT, respectively [43–45]. The ion-gel is a solid electrolyte, and its mobile ions ([EMIM]^+^, [TFSI]^−^) can be regarded as neurotransmitters, so that the process of the ion migration/accumulation is critical for the mimicking of biological synapses [46–48]. To illustrate the dynamics of the ion migration/accumulation, the capacitive properties of the ion-gel are investigated by impedance spectroscopy through a typical metal–insulator-metal (MIM) capacitor model (Fig. S1a) [49–51]. The capacitance parameters of the ion-gel are measured by loading an AC voltage with frequency from 20 Hz to 20 MHz (Fig. S1b-d). Figure S1b shows that the capacitance of the ion-gel exceeds 10 μF cm^−2^ at a low frequency of 20 Hz, and is still higher than 1 μF cm^−2^ at a high frequency of 0.2 MHz. This is due to the rapid formation of electric double layers (EDLs) at the electrode/ion-gel interface by the mobile ions, which indicates that the polymer network in the ion gel does not inhibit the migration speed of the ions. The phase angle of the ideal capacitor is −90°, while the phase angle of the ideal resistor is 0°, that is, the phase angle is −45°, which is the transition point from capacitance characteristic to resistance characteristic. Figure S1c shows the relationship between the corresponding phase angle of ion-gel capacitor and the loaded frequency. When the frequency is less than 0.06 MHz, the ion-gel shows significant capacitive behavior, but gradually changes to a resistive behavior with the increase in frequency. The Nyquist plots show the real and imaginary parts of the impedance of the ion-gel, which is observed that the slope is always positive (Fig. S1d). The results demonstrate that the ion-gel can form EDLs capacitor with good charge storage capacity, which enables to tune the semiconductor channel carriers transport of the OTFT.

Moreover, P3HT can quickly form high-quality P3HT nanofibrils (P3HT-NF) by simple heating and cooling process [52]. Specifically, P3HT is first dissolved in *m*-xylene solvent at 60 °C, and then naturally cooled to room temperature (Fig. S2a, bright orange). Subsequently, the solution is fully mixed with *m*-xylene diluted PDMS and deposited at  − 15 ℃ for 30 min, which make it have the ability to tolerated mechanical deformation (Fig. S2b, dark purple). Interestingly, the P3HT-NF/PDMS semiconductor solution can remain uniformly dispersed throughout the heating and cooling process. Previous studies have shown that the surface roughness of thin film formed by semiconductor solution has a significant impact on the performance of electronic devices [53, 54]. To study the surface roughness, the surface morphology of the P3HT-NF/PDMS semiconductor film is characterized by AFM. The P3HT-NF/PDMS semiconductor film is obtained by spin-coating the P3HT-NF/PDMS semiconductor solution on a thin flexible PET film and annealing. Figure S2c displays the surface morphology of the P3HT-NF/PDMS semiconductor film with a scanning area of 5 × 5 μm^2^. The result shows that the surface morphology of the P3HT-NF/PDMS semiconductor film is quite smooth and uniform, thereby reducing the scattering of carriers and the trapping of interface charges, which is very beneficial for improving the performance of the TAS.

To evaluate the performance of the TAS, the electrical characteristics of the OTFT were first studied. Figure 1d displays the double-sweep transfer characteristics of the OTFT when the source-drain voltage (*V*_*d*_) is kept at  − 0.5 V and the gate voltage (*V*_*g*_) is between 2 and  − 2 V. It is seen that the OTFT has typical *P*-type transistor behavior, and exhibits an obvious hysteresis window. In addition, the double-sweep transfer characteristics of three repeated sweeps have no significant change. This is mainly due to the intrinsic characteristics of the P3HT-NF/PDMS semiconductor and the migration of ions in the ion-gel. The output characteristics of the OTFT under different *V*_*g*_ are shown in Fig. 1e. Under the action of larger *V*_*g*_, the *I*_*d*_ of the OTFT increase significantly, and the *I*_*d*_ increases linearly at low *V*_*d*_, while the *I*_*d*_ tends to saturate gradually at high *V*_*d*_. The results demonstrate that the semiconductor layer has good Ohmic contact with the source-drain electrodes and highly efficient coupling with the ion-gel. The mobility (*µ*) of the OTFT in the linear can be calculated by the following equation:

1 $$I_{d} = \frac{W}{L} \cdot \mu C(V_{g} - V_{t} ) \cdot V_{d}$$

where *W* is the channel width, *L* is the channel length, *C* is the specific capacitance of the ion-gel, *V*_*t*_ is the threshold voltage. By extracting the parameters in Fig. 1d, it can be calculated that the *µ* is 1.39 cm^2^ V^−1^ s^−1^. To examine electrical characteristics of the OTFT under bending strain (Fig. S3a), the double-sweep transfer characteristics under different bending radius in tensile and compressive strain states are measured (Fig. S3b–c). Based on these transfer curves, the corresponding *µ* is calculated, as shown in Fig. 1f. The double-sweep transfer characteristics of the OTFT under different *V*_*d*_ are shown in Fig. S3d. In addition, the electrical characteristics of the OTFT after different number of tensile and compressive strain cycles with a bending radius of 20 mm are further measured, the results are shown in Fig. S3e–g. It can be seen that the electrical characteristics of the OTFT change very little and can be ignored. This is because each material used to prepare the OTFT has excellent mechanical flexibility, which provides a reliable guarantee for the development of the TAS.

### Working Mechanism of the TAS

**Fig. 2:**
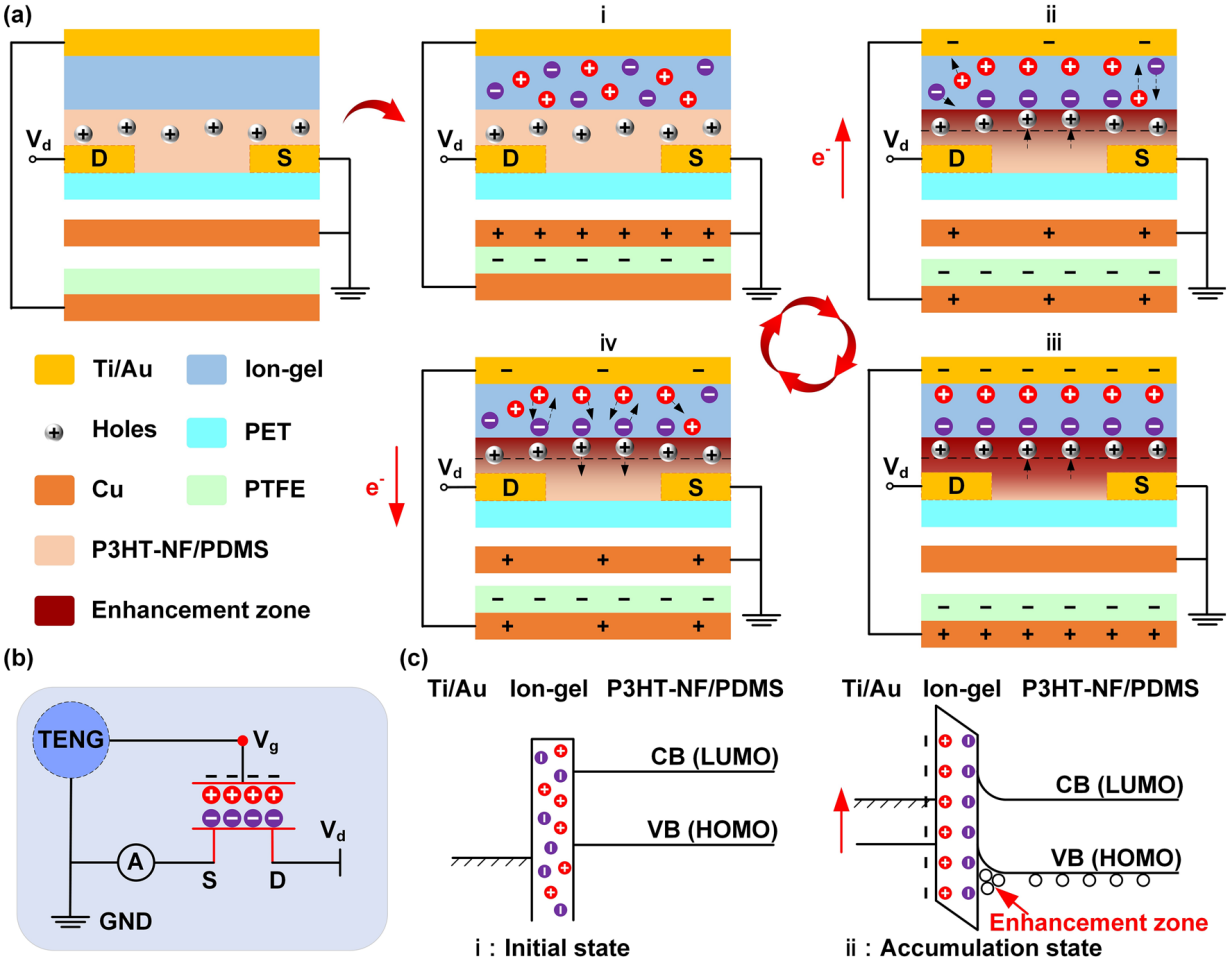
Working mechanism of the TAS. **a** Schematic working principle of the TAS. **b** Equivalent circuit diagram of the TAS. **c** Energy band diagram of the TAS

The working mechanism of the TAS is based on the coupling effects of the electric double layer (EDL) OTFT and the triboelectric potential generated by the external contact electrification, which is schematically shown in Fig. 2. The Cu film is selected as the externally moving friction layer, which is combined with the PTEF/Cu structure to form a TENG in contact-separation mode. The Cu film is connected to the source electrode of the OTFT and grounded, the Cu electrode of the PTEF/Cu structure is connected to the gate electrode of the OTFT, and the drain electrode of the OTFT is connected to a power supply. In the initial state, the Cu film is in full contact with the PTFE friction layer, which induces equal positive and negative charges on the surfaces of the Cu film and PTFE film due to the electrostatic induction, respectively (Fig. 2a(i)). At this moment, no electrons are transported from the Cu electrode to the gate of the OTFT, so that the anions and cations in the ion-gel are randomly distributed and the holes concentration in the P3HT-NF/PDMS semiconductor channel keeps the original value. It can also be seen from the corresponding energy band diagram that the vacuum energy level of the Ti/Au electrode and the P3HT-NF/PDMS semiconductor is the same and the conduction band and valence band are in the flat-band situation (Fig. 2c(i)). With the gradual separation of the Cu film from PTFE friction layer, the Cu electrode gradually loses electrons and induces the triboelectric potential to maintain the electrostatic balance. The gate of the OTFT thus receives the electrons, that is, a negative gate voltage is applied to the gate, so that the anions and cations in the ion-gel begin to migrate (Fig. 2a(ii)). The anions begin to migrate to the ion-gel/P3HT-NF/PDMS semiconductor interface, while the cations begin to move to Ti/Au/ion-gel. When the Cu film is separated to a certain distance, more electrons flow from the Cu electrode to the gate of OTFT and the negative gate voltage reaches the maximum value, making the anions and cations gather at the ion-gel/P3HT-NF/PDMS semiconductor interface and the Ti/Au/ion-gel interface respectively, thus forming the EDLs (Fig. 2a(iii)). This process results in the increase in the holes concentration in the P3HT-NF/PDMS semiconductor channel and generates a directional moving current between the source and drain electrodes (i.e., *I*_*d*_). The corresponding energy band diagram is shown in Fig. 2c(ii), the holes concentration of the semiconductor channel increases due to the accumulation of anions at the ion-gel/P3HT-NF/PDMS semiconductor interface, which forms an enhancement zone and bends the energy band upward. As the Cu film gradually returns to the initial state, electrons return from the gate to the Cu electrode, and the negative gate voltage gradually recovers to 0 V, so that previous accumulated anions and anions migrate again (Fig. 2a(iv)). In this process, the holes concentration in the P3HT-NF/PDMS semiconductor channel gradually reduces, while the source-drain current gradually decreases and finally returns to the initial value. The working mechanism of the TAS can be equivalent to the circuit in Fig. 2b. Replacing the gate voltage of the OTFT by the triboelectric potential generated by the external contact electrification can not only reduce the power consumption of the device, but also can establish an active interaction mechanism between the external stimulus and the device.

### Electrical Characteristics of the TAS

**Fig. 3:**
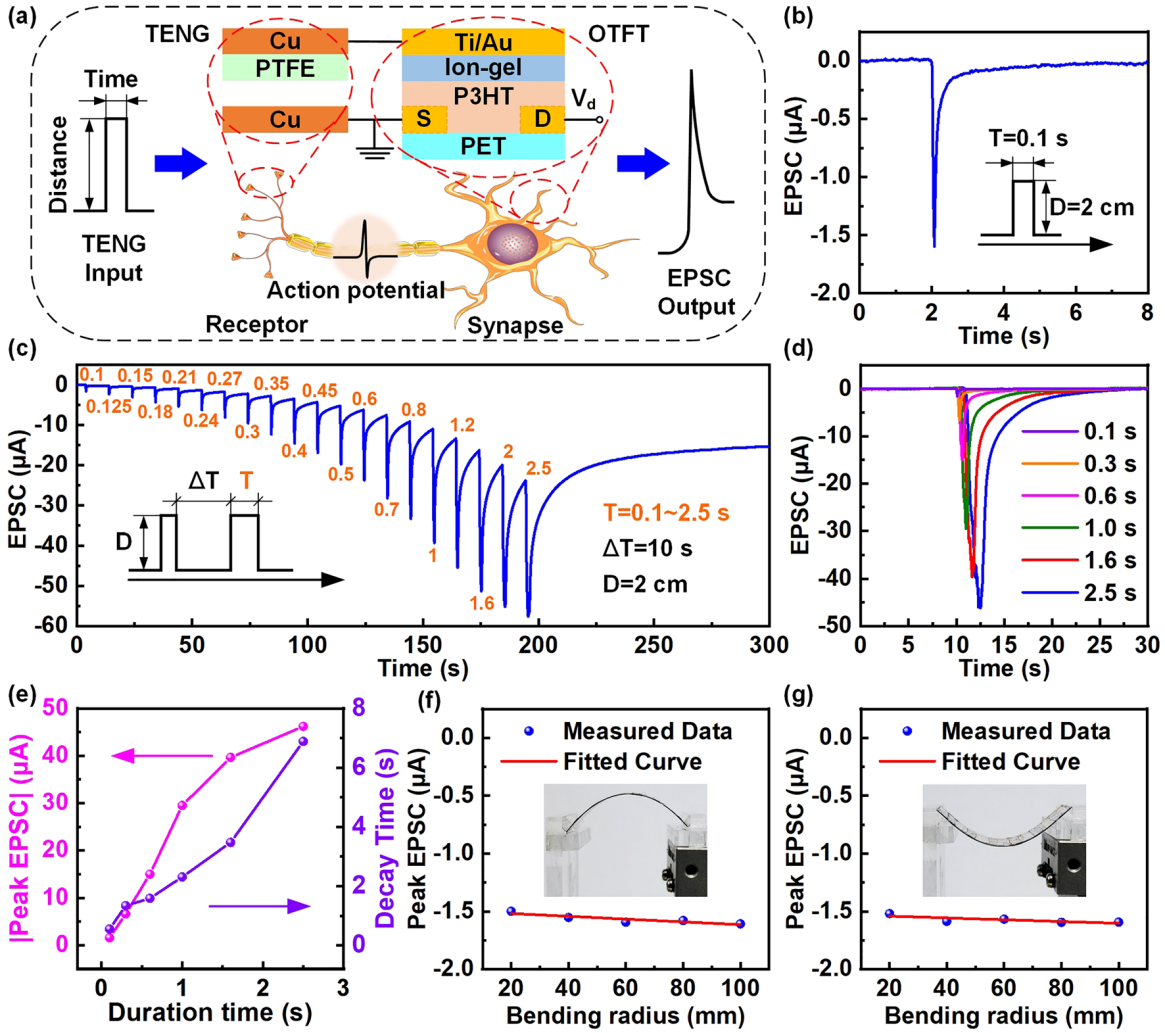
Basic synaptic characteristics of the TAS. **a** Schematic illustration of the analogy between biological afferent neuron and the TAS device. **b** EPSC response of the TAS device under single mechanical stimuli (*T* = 0.1 s, *D* = 2 cm). **c** EPSC response of the TAS device under the continuous action of single mechanical stimuli with different duration time at fixed interval (*T* = 0.1 ~ 2.5 s, Δ*T* = 10 s, *D* = 2 cm). **d** Comparison of the EPSC response of the TAS under different duration time of single mechanical stimuli. **e** EPSC peak absolute value and decay time of the TAS under different duration time of single mechanical stimuli. **f** The effect of different bending radius in tensile strain states on EPSC peak value of the TAS under single mechanical stimuli (*T* = 0.1 s, *D* = 2 cm). **g** The effect of different bending radius in compressive strain states on EPSC peak value of the TAS under single mechanical stimuli (*T* = 0.1 s, *D* = 2 cm)

To demonstrate the bioinspired neurosensory behavior of the TAS, the electrical characteristics of the coupling of the EDL OTFT and the triboelectric potential generated by the external contact electrification were investigated. Figure 3a shows the schematic illustration of the analogy between biological afferent neuron and the TAS device. The TENG formed by the Cu film and the PTEF/Cu part is considered as biological tactile receptor, while the EDL OTFT is regarded as biological synapse. Specifically, the TENG can realize contact-separation under mechanical stimuli, and obtain relevant external force information according to the generated triboelectric potential signal. The triboelectric potential is regarded as a presynaptic signal and acts on the ion-gel-gate voltage of the EDL OTFT to induce the migration of anions and anions in the ion-gel to form the EDL, so as to effectively tune the carriers transport of the semiconductor channel to form a postsynaptic current. During the whole measure of the electrical characteristics, the displacement distance (*D*) of TENG is 2 mm, and the *V*_*d*_ applied to the EDL OTFT is 0.5 V. A typical EPSC of the TAS activated by single mechanical stimuli with a duration time of 0.1 s is shown in Fig. 3b. It can be observed that the EPSC rapidly rises to the peak current (1.6 μA), and then gradually decays back to the initial current. This process is similar to an expression of excitatory behavior in biological synapses, which is a basic characteristic of neurosensory behavior and can also be considered as a SM mode in the multistore memory model. Moreover, the EPSC output response of the TAS continuously activated by single mechanical stimuli with different duration time at fixed interval was also studied, as shown in Fig. 3c. The result shows that the EPSC peak current constantly increased with the increase in the duration time. This is mainly attributed to the dynamic behavior of ions migration-relaxation [55]. The anions induced by the previous mechanical stimuli does not have enough time to migrate before the arrival of the next mechanical stimuli, which leads to the accumulation of more and more anions at the ion-gel/P3HT-NF/PDMS semiconductor interface, and finally makes the output peak of the EPSC continue to increase. To clarify the effect of the duration time on the EPSC, it is necessary to explore the output response of the TAS under different duration time of single mechanical stimuli. As can be observed from Fig. 3d, the output peak of the EPSC increases with the increase in the duration time, while the slower of the decay time. This slow decay behavior can be fitted with a stretched-exponential decay model, which is expressed as [56]:

2 $$I(t) = (I_{{{\text{peak}}}} - I_{\infty } ) \cdot \le \exp \left[ {\left( {\frac{{ - (t - t_{0} )}}{\tau }} \right)^{\beta } } \right] + I_{\infty }$$

where *τ* is the decay time, *I*_peak_ is the peak value of the EPSC, *I*_*∞*_ is the final value after the decay of EPSC, *t*_*0*_ is the end time of mechanical stimuli, *β* is a correction factor between 0 and 1. The results obtained by fitting according to relevant experimental data and formulas are shown in Fig. 3e, and the good fitting curves are shown in Fig. S3.

Noticeably, excellent mechanical flexibility of the TAS is crucial to meet the application requirements in curved and dynamic surfaces. Figure 3f, g displays the effect of different bending radius in tensile and compressive strain states on EPSC peak value of the TAS under single mechanical stimuli with a duration time of 0.1 s, respectively. More measure results with different duration time are shown in Fig. S4. The results show that the TAS is less sensitive to tensile and compressive strains under different bending radius, and the change of the EPSC peak is very little and negligible. Even if the bending radius has been reduced to 20 mm, the output characteristics of the TAS still show significant stability. These findings indicate that the tensile and compressive strains under different bending radius have no effect on the EPSC characteristics of the TAS.

**Fig. 4:**
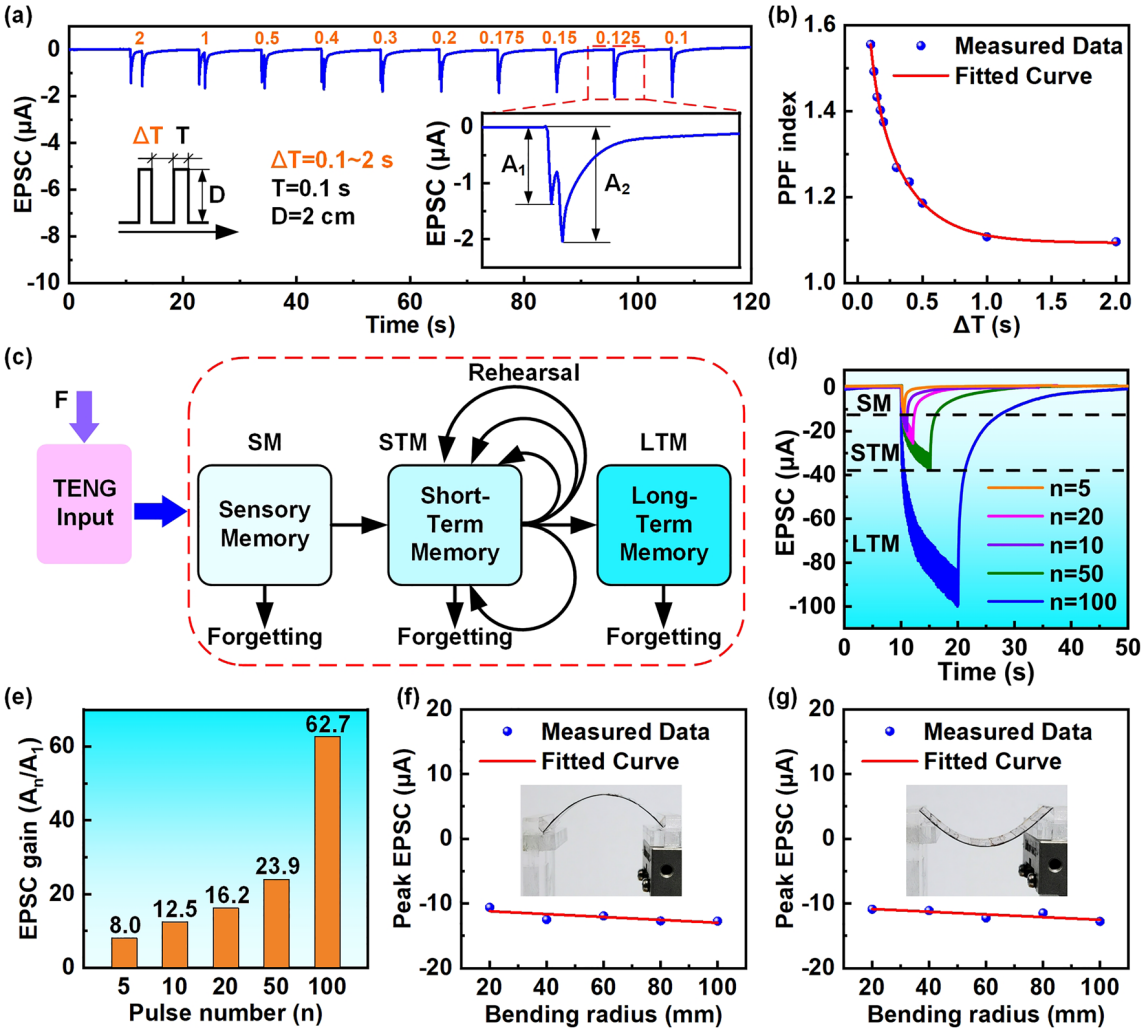
Typical synaptic plasticity of the TAS. **a** EPSC responses of a series of the TAS under paired mechanical stimuli with different interval times (Δ*T* = 0.1 ~ 2.5 s, *T* = 0.1 s, *D* = 2 cm). The inset is an enlarged view of the EPSC response of the TAS under paired mechanical stimuli with an interval time of 0.125 s. **b** The effect of the paired mechanical stimuli with different interval times on the PPF index of the TAS (PPF index is defined as* A*_2_/*A*_1_). **c** Multistore memory model with TENG as input. **d** EPSC response of the TAS under multiple continuous mechanical stimuli (*T* = 0.1 s, Δ*T* = 0.1 s, *D* = 2 mm). **e** The effect of the multiple continuous mechanical stimuli on the EPSC gain of the TAS (EPSC gain is defined as* A*_n_/A_1_). **f** The effect of different bending radius in tensile strain states on EPSC peak value of the TAS under 5 times of mechanical stimuli (*T* = 0.1 s, Δ*T* = 0.1 s, *D* = 2 mm). **g** The effect of different bending radius in compressive strain states on EPSC peak value of the TAS under 5 times of mechanical stimuli (*T* = 0.1 s, Δ*T* = 0.1 s, *D* = 2 mm)

Generally, the excitatory behavior of biological synapses can be further enhanced by applying multiple stimuli signals, that is, typical synapse plasticity. The TAS should also have similar synapse behavior. Therefore, the output behavior of the TAS under paired mechanical stimuli was further studied. Figure 4a displays the EPSC responses of the TAS under paired mechanical stimuli with different interval times, and the inset is an enlarged view of the EPSC response of the TAS under paired mechanical stimuli with an interval time of 0.125 s. It is seen that the EPSC peak value (*A*_2_) caused by the second stimuli is obviously greater than that (*A*_1_) caused by the first stimuli. This phenomenon is a typical PPF behavior and belongs to a kind of short-term synaptic plasticity, which is of great significance for the brain-inspired computing [57, 58]. This facilitation degree is closely related to the interval time between paired mechanical stimuli and can be evaluated by the PPF index, which is defined as *A*_2_/*A*_1_. As seen in Fig. 4b, the PPF index decreases with the increase in interval time, and this decay process can be well fitted with a double exponential decay function [57]:

3 $${\text{PPF}}\;{\text{index}} = A + B_{1} \cdot \exp \left( { - \Delta t/\tau_{1} } \right) + B_{2} \cdot \exp \left( { - \Delta t/\tau_{2} } \right)$$

where *A* is a constant, *Δt* is the interval time between paired mechanical stimuli, *B*_1_ and *B*_2_ are the initial facilitation amplitudes, *τ*_1_ and *τ*_2_ are the relaxation time of the rapid and the slow decay phases, respectively. By fitting, the values of *τ*_1_ and *τ*_2_ are 57 and 298 ms, respectively, which is consistent with the time scale in biological synapses.

Inspired by the multistore model of memory [59, 60], the simplified memory model corresponding to the bioinspired neurosensory behavior of the TAS under mechanical stimuli is shown in Fig. 4c. The simplified memory model explains the hierarchical memory process of the TAS from SM to STM and LTM under external mechanical stimuli. To verify the hierarchical memory process, the EPSC response of the TAS under multiple continuous mechanical stimuli was studied, as shown in Fig. 4d. When 5 times of the mechanical stimuli (*T* = 0.1 s, Δ*T* = 0.1 s, *D* = 2 mm) are performed, the EPSC of the TAS gradually increases to a peak value of 12.8 μA, and then decay to the initial value with the end of the mechanical stimuli. As the number of the mechanical stimulus increases, the output EPSC peak value of the TAS increases continuously. Until the number of the mechanical stimulus increases to 100, the highest EPSC peak value of the TAS can increase to 99.9 μA, and it can be observed that the decay of the EPSC return to the initial value until about 50 s. In addition, the EPSC gain can further quantify the reinforcement process of the hierarchical memory, which is defined as *A*_n_/*A*_1_, and the results are shown in Fig. 4e. It is seen that the EPSC gain is closely related to the pulse number of the mechanical stimuli, and increases with the increase in the pulse number. The results demonstrate that the hierarchical memory process of the TAS from SM to STM and LTM can be achieved by increasing the pulse number of external mechanical stimuli.

Furthermore, the effect of different bending radius in tensile and compressive strain states on the typical synaptic plasticity of the TAS was studied, respectively. Figure S5 shows the effect of different bending radius in tensile and compressive strains on the PPF behavior of the TAS under paired mechanical stimuli. It is seen that the PPF index of the TAS still decreases with the increase in interval time, and their decay process can also be well fitted with the double exponential decay function (Figs. S6 and S7). These results indicate that the TAS still exhibits good PPF behavior even under tensile and compressive strains with a bending radius of 20 mm. Figure 4f, g displays the effect of different bending radius in tensile and compressive strain states on EPSC peak value of the TAS under 5 times mechanical stimuli (*T* = 0.1 s, Δ*T* = 0.1 s, *D* = 2 mm), respectively. It is obvious that the EPSC peak value of the TAS changes very little, which indicates that the bending radius has negligible effect on the output characteristics of the TAS under compressive and tensile strain states. The measure results under more mechanical stimuli number are shown in Fig. S8. It can be seen that the tensile and compressive strains under different bending radius are also less sensitive to the EPSC peaks of the TAS under the action of other multiple consecutive mechanical stimulus, and the EPSC gain changes are also small and can be ignored. Thus, this further indicate that the TAS still exhibits good hierarchical memory process under tensile and compressive strains with different bending radius. The above studies demonstrate that the TAS has excellent mechanical flexibility and can realize typical synaptic behavior under different bending modes and radius, which has broad application prospects in the field of artificial limbs, robotics, and bionics in future.

**Fig. 5:**
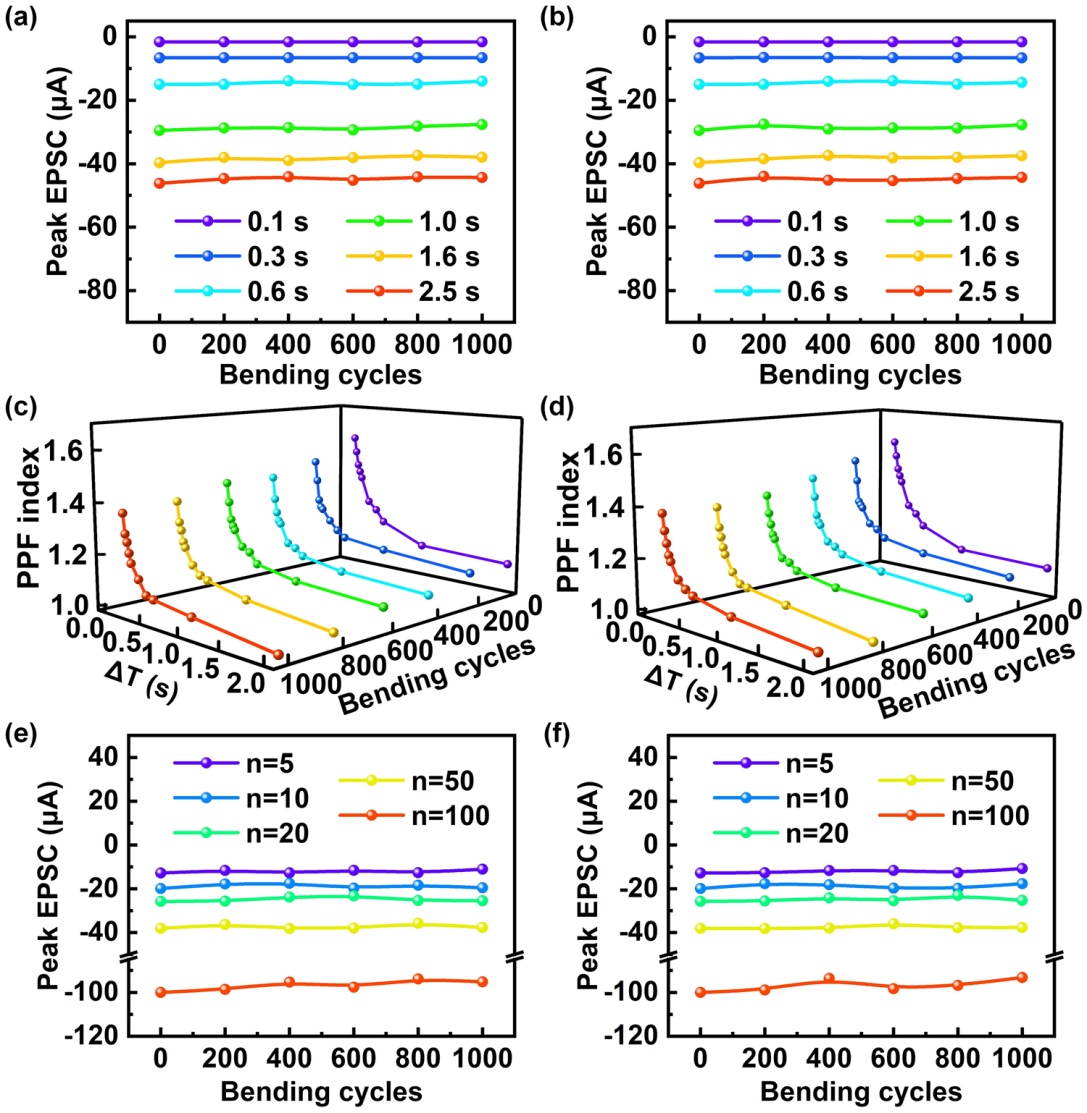
Stability and durability of the TAS. **a** EPSC peak changes of the TAS under single mechanical stimulus with different duration times after number of tensile strain cycles with a bending radius of 20 mm. **b** EPSC peak changes of the TAS under single mechanical stimulus with different duration times after number of compressive strain cycles with a bending radius of 20 mm. **c** PPF index changes of the TAS under paired mechanical stimulus with different duration times after number of tensile strain cycles with a bending radius of 20 mm. **d** PPF index changes of the TAS under paired mechanical stimulus with different duration times after number of compressive strain cycles with a bending radius of 20 mm. **e** EPSC peak changes of the TAS under number of mechanical stimuli with a duration time of 0.1 s after number of tensile strain cycles with a bending radius of 20 mm. **f** EPSC peak changes of the TAS under number of mechanical stimuli with a duration time of 0.1 s after number of compressive strain cycles with a bending radius of 20 mm

To evaluate the stability and durability of the TAS, the output characteristics of the TAS after tensile and compressive strain cycles with a bending radius of 20 mm were further investigated, as shown in Fig. 5. It is seen clearly that the output characteristics of TAs have no obvious change after continuous bending tensile and compressive strain cycles. Figure 5a and b shows the EPSC peak changes formed by single mechanical stimulus with different duration times after different tensile and compressive strain cycles of the TAS, respectively. It can be seen that the number of cycles have a quite small effect on the EPSC peak values of the TAS. For example, the EPSC peak value of the TAS decreases by only ~ 1.7% ( 46.2 t–45.4 μA) even after 1000 tensile strain cycles of continuous bending. For the compressive strain cycles, the EPSC peak value of the TAS decreases by only ~ 3.2% ( 46.2 t–44.7 μA). Figure 5c, d shows the PPF index changes formed by paired mechanical stimulus with different interval times after different tensile and compressive strain cycles, respectively. Obviously, the PPF index of the TAS before and after the strain cycle has similar trends, and their decay process can also be well fitted with the double exponential decay function (Figs. S9 and S10). Figure 5e, f shows the EPSC peak changes formed by multiple mechanical stimulus with a duration time of 0.1 s after tensile and compressive strain cycles, respectively. The corresponding EPSC gain changes are shown in Fig. S11. Compared with the previously reported studies (Table S1), the TAS still exhibits excellent synaptic behavior even after 1000 bending cycles of tensile and compressive strains, which is more helpful to promote its practical applications in artificial limbs, robotics, and bionics.

**Fig. 6:**
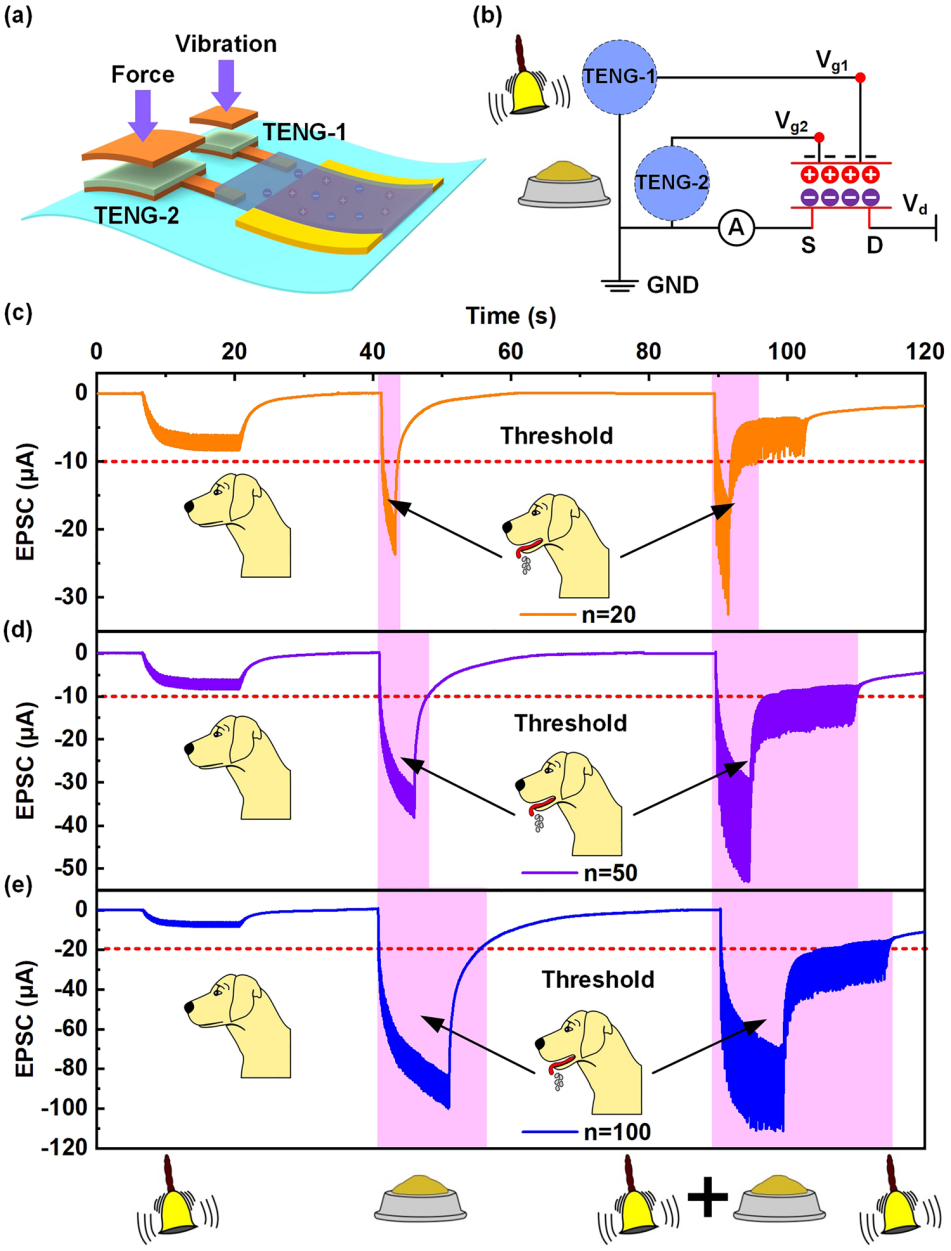
A simulation experiment of associative memory in Pavlov’s dog. **a** Schematic illustration of the TAS device under force and vibration dual TENG mode. **b** Equivalent circuit diagram of the TAS in dual TENG mode and analogy of Pavlov’s dog. **c-e** EPSC response of the TAS device changes when the training time is 20, 50 and 100

In physiology, conditioned reflex is a typical associative learning form, which is gradually formed through acquired learning [61, 62]. Specifically, Pavlov’s dog is a famous classical conditioned reflex experiment, which uses “bell” as a neutral stimulus and “food” as an unconditioned stimulus. The “salivation” response of the dog cannot be triggered by neutral stimuli alone, but after multiple combination training of neutral stimuli and unconditioned stimuli, neutral stimuli can also make it produce “salivation” response. However, the postsynaptic current generated by artificial synapses can be used to mimic the “salivation” response. To demonstrate the TAS can mimic the conditioned reflex experiment, force and vibration stimuli is regarded as neutral and unconditioned stimuli, respectively. Therefore, the associative learning behavior of TAS is to further study through the improved structure, as shown in Fig. 6a. Because the ion-gel can be controlled by multiple electrodes, the output characteristics of the TAS can be tuned through the triboelectric potential generated by two different TENGs. The corresponding equivalent circuit diagram is shown in Fig. 6b. In this simulation experiment, TENG-1 actuated by vibration is regarded as the input source of “bell”, while TENG-2 actuated by force is regarded as the input source of “food”. Under the action of “bell”, the output EPSC peak of the TAS is 8.5 μA. Before learning and training, “bell” as an input source cannot cause the “salivation” response. Therefore, the EPSC peak at 10 μA is considered as the threshold for the “salivation” response. However, when 20 “food” stimuli are performed, the TAS output EPSC peaked can reach 25.8 μA, which imply that the “salivation” response is triggered (Fig. 6c). When 20 “bell” and “food” stimuli are applied simultaneously, the output EPSC peak of the TAS far exceed the threshold. It is worth noting that when the “food” stimuli is removed, the output EPSC of the TAS did not immediately drop to the threshold, but remained for a period of time. The results indicate that after learning and training, a link between “bell” and “food” has been established, which makes the TAS also produce “salivation” response under the effect of “bell”. Further, 50 and 100 times of learning and training are performed, respectively, as shown in Fig. 6d, e. It can be seen that the longer the training times increase, the longer the “salivation” response is maintained. The experimental results demonstrate that the TAS can effectively mimic the conditioned reflex, which will provide effective help for the future research on motion feedback in bionics.

## Conclusions

In summary, a flexible TAS coupled by triboelectric potential and EDL OTFT was reported, which can successfully realize the bioinspired neurosensory behavior. The triboelectric potential generated by the external contact electrification is used as the ion-gel-gate voltage of the OTFT to tune the channel carriers transport through the migration/accumulation of ions, thus establishing an active interaction mechanism. The relationship between the output characteristics of the TAS and external stimuli is systematically investigated. A series of synaptic behaviors of the TAS actuated by external stimuli were successfully demonstrates, such as EPSC, PPF, and the hierarchical memory process from SM to STM and LTM. This is mainly attributed to the dynamic behavior of ions migration-relaxation. The TAS exhibits excellent mechanical flexibility, which can still remain stable synaptic behaviors under the tensile and compressive strain states with a bending radius of 20 mm. Moreover, the TAS still exhibits excellent durability even after 1000 bending tensile and compressive strain cycles with a bending radius of 20 mm. Finally, Pavlovian conditioning has been successfully mimicked by applying force and vibration as food and bell, respectively. This work demonstrates a bioinspired flexible artificial synapse that will help to facilitate the development of artificial afferent nervous systems, which is great significance to the practical application of artificial limbs, robotics, and bionics in future.

### Supplementary Information

Below is the link to the electronic supplementary material.

Supplementary file1 (PDF 1920 KB)
