# Supplementary material for: A Flexible Tribotronic Artificial Synapse with Bioinspired Neurosensory Behavior
Source: Nanomicro Lett. 2022 Dec 29;15:18. doi: 10.1007/s40820-022-00989-0 (PMC9800681; doi:10.1007/s40820-022-00989-0)
Supplement: Supplementary file 1 — Supplementary file1 (PDF 1920 KB) [file 40820_2022_989_MOESM1_ESM.pdf]

Supporting Information for

A Flexible Tribotronic Artificial Synapse with Bioinspired Neurosensory Behavior

Jianhua Zeng[1, 2, †], Junqing Zhao[1, 3, †], Tianzhao Bu[1, 3], Guoxu Liu[1, 3], Youchao Qi[1, 3], Han Zhou[1, 2], Sicheng Dong[1, 3], Chi Zhang[1, 2, 3, *]

[1] CAS Center for Excellence in Nanoscience, Beijing Key Laboratory of Micro-nano Energy and Sensor, Beijing Institute of Nanoenergy and Nanosystems, Chinese Academy of Sciences, Beijing 101400, P. R. China

[2] Center on Nanoenergy Research, School of Physical Science and Technology, Guangxi University, Nanning, 530004, P. R. China

[3] School of Nanoscience and Technology, University of Chinese Academy of Sciences, Beijing 100049, P. R. China

[†]Jianhua Zeng and Junqing Zhao contributed equally to this work.

*Corresponding author. E-mail: czhang@binn.cas.cn (Chi Zhang)

Supplementary Figures and Tables

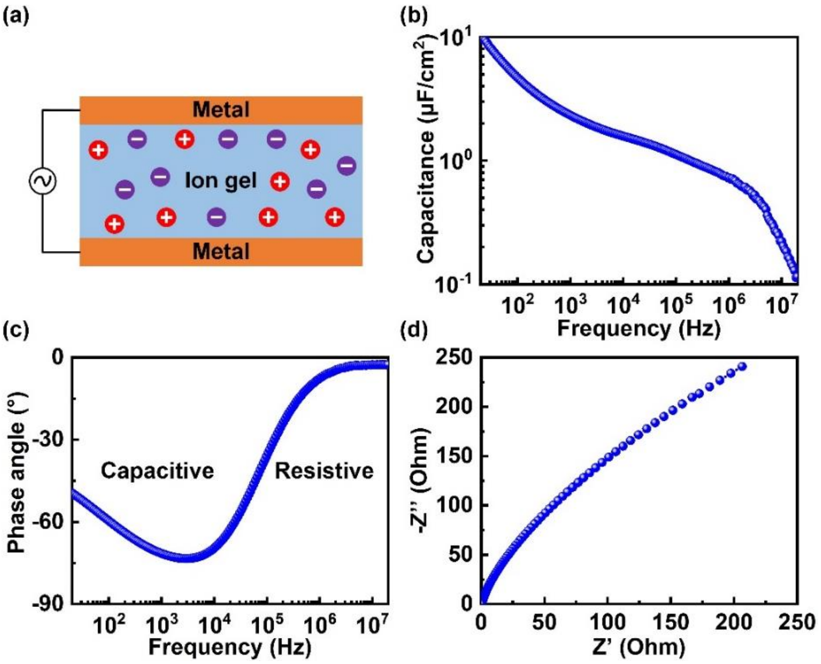

Fig. S1 Impedance analysis of the ion-gel dielectric film. (**a**) Typical MIM structure diagram of the ion-gel dielectric film. (**b**) The functional relationship between the capacitance of the ion-gel dielectric film and the frequency of applied AC. (**c**) The functional relationship between the phase angle of the ion-gel dielectric film and the frequency of applied AC. (**d**) Nyquist plot of the ion-gel dielectric film

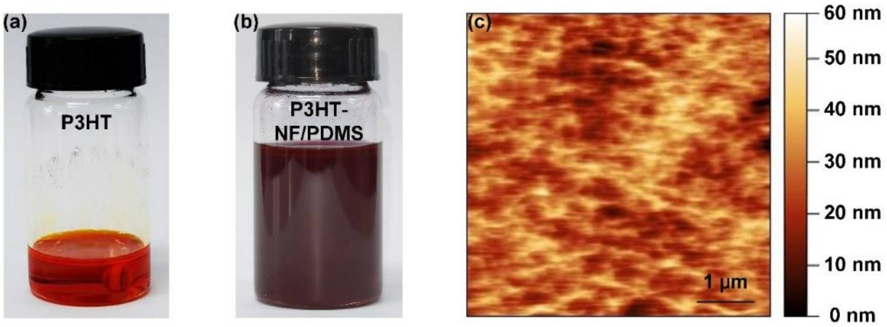

Fig. S2 Characterization of the P3HT-NF/PDMS semiconductor. (**a**) Optical image after complete dissolution of P3HT. (**b**) Optical image of the P3HT-NF/PDMS semiconductor solution. (**c**) The AFM morphology of the P3HT-NF/PDMS semiconductor film

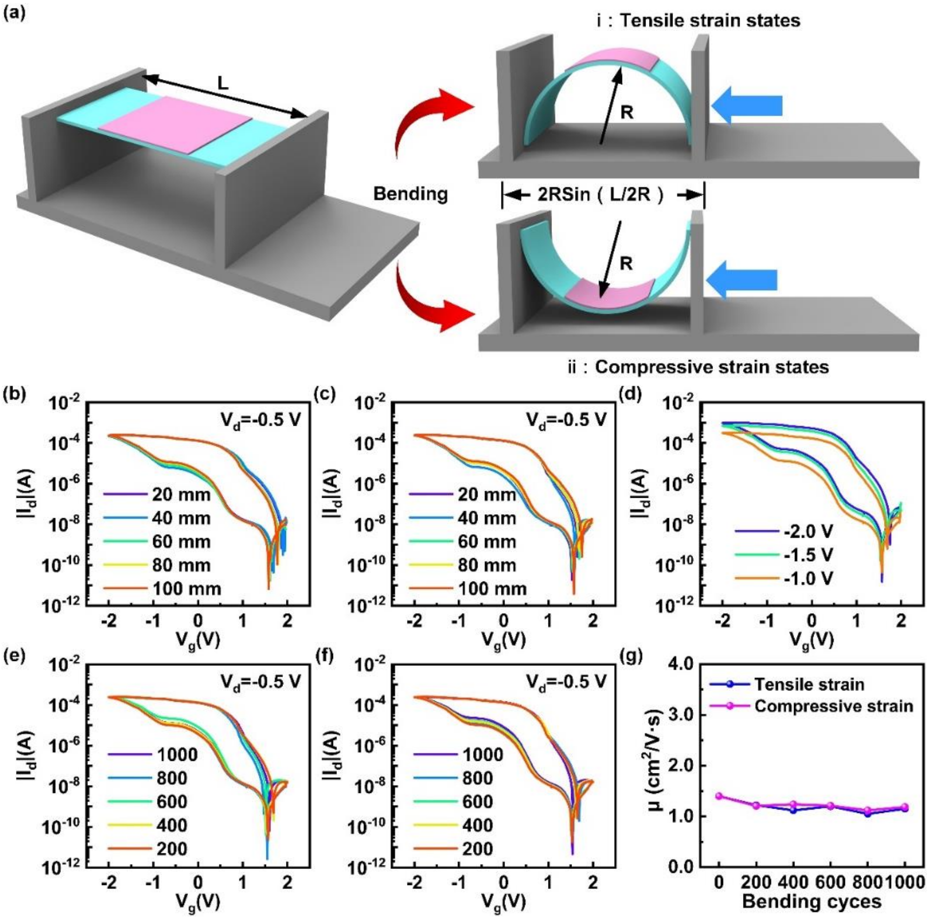

Fig. S3 The bending process diagram and electrical characteristics of the OTFT. (**a**) Schematic illustration of the OTFT in tensile and compressive strain states, respectively. (**b**) Double-sweep transfer characteristics of the OTFT under different bending radius in tensile strain states. (**c**) Double-sweep transfer characteristics of the OTFT under different bending radius in compressive strain states. (**d**) Double-sweep transfer characteristics of the OTFT under different V_d. (**e**) Double-sweep transfer characteristics of the OTFT after different number of tensile strain cycles with a bending radius of 20 mm. (**f**) Double-sweep transfer characteristics of the OTFT after different number of compressive strain cycles with a bending radius of 20 mm. (**g**) The mobility of the OTFT after different number of tensile and compressive strain cycles with a bending radius of 20 mm

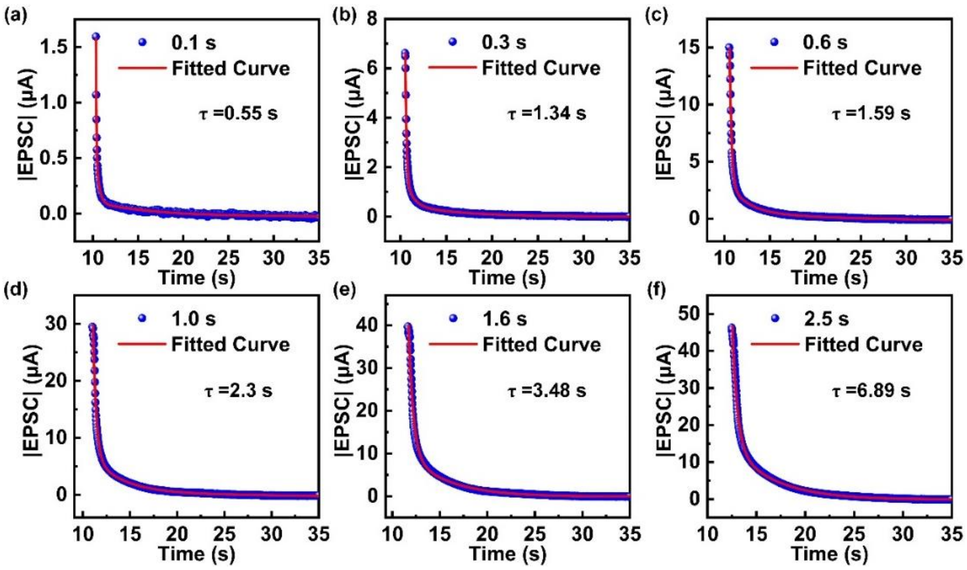

Fig. S4 The fitting of the EPSC decay behavior with stretched-exponential decay model. (**a-f**) The fitting of the EPSC decay behavior with stretched-exponential decay model under single mechanical stimuli with different duration time (0.1 s, 0.3 s, 0.6 s, 1.0 s, 1.6 s and 2.5 s)

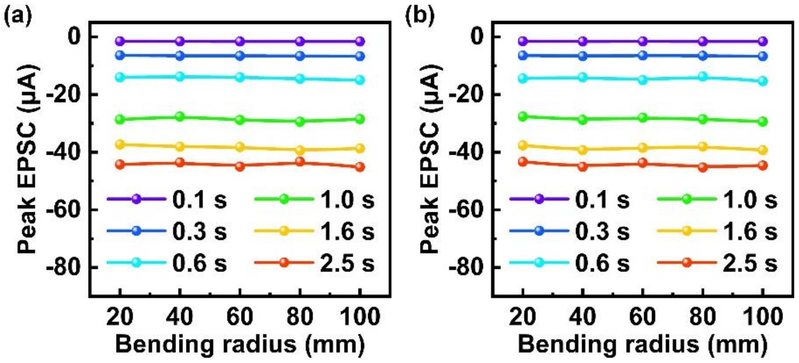

Fig. S5 The effect of different bending radius on EPSC peak value of the TAS under single mechanical stimuli with different duration time. (**a**) The effect of different bending radius in tensile strain states on EPSC peak value of the TAS under single mechanical stimuli with different duration time. (**b**) The effect of different bending radius in compressive strain states on EPSC peak value of the TAS under single mechanical stimuli with different duration time

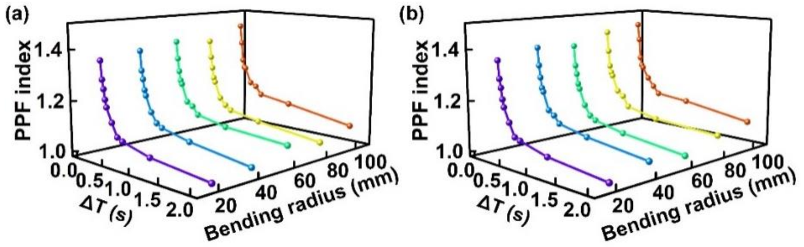

Fig. S6 The effect of different bending radius on PPF index of the TAS under paired mechanical stimuli with different interval time. (**a**) The effect of different bending radius in tensile strain states on PPF index of the TAS under paired mechanical stimuli with different interval time. (**b**) The effect of different bending radius in compressive strain states on PPF index of the TAS under paired mechanical stimuli with different interval time.

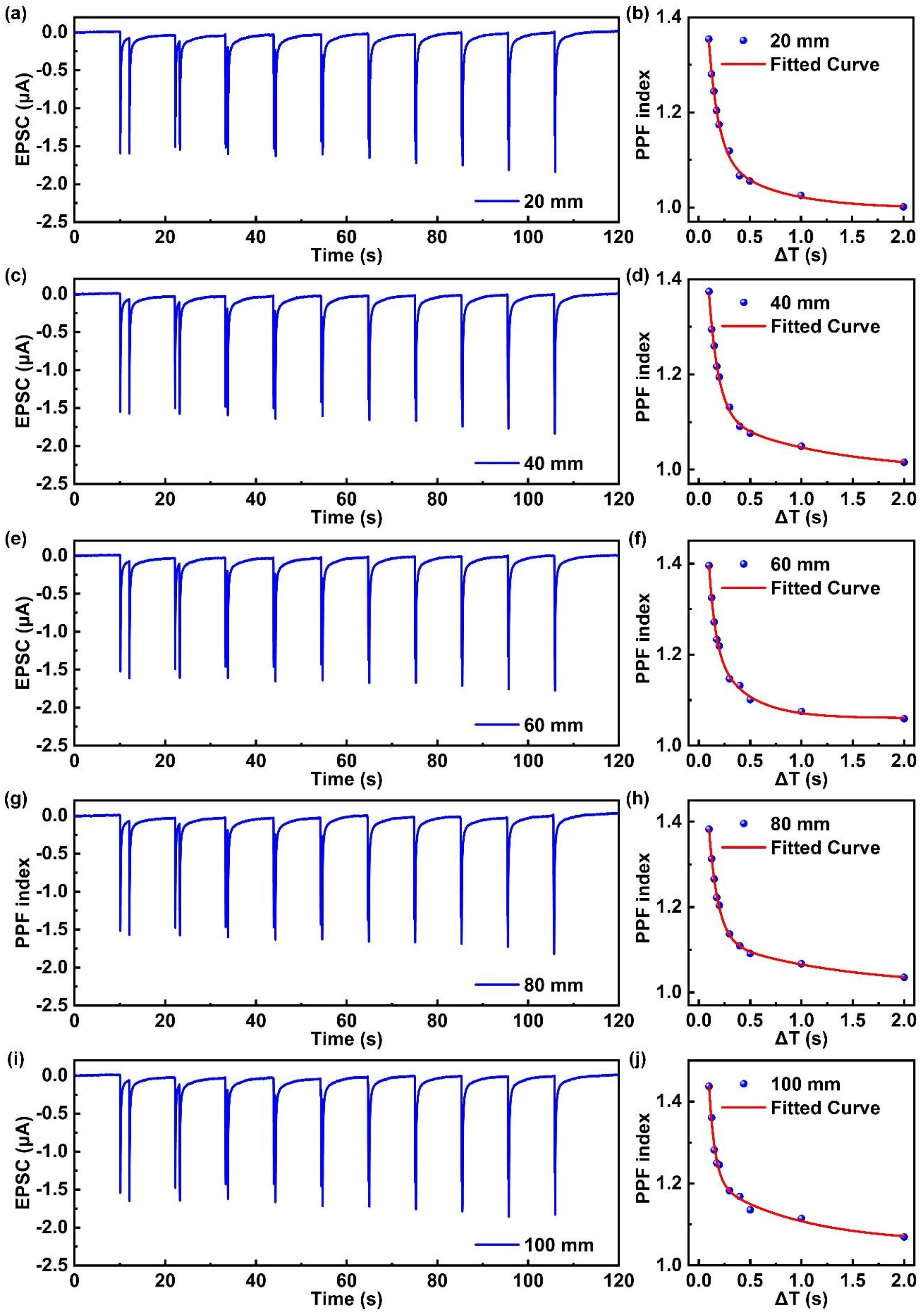

Fig. S7 EPSC and PPF index response of the TAS under different bending radius in tensile strain states. (**a**), (**c**), (**e**), (**g**) and (**i**) The EPSC response of the TAS under different bending radius (20, 40, 60, 80 and 100 mm) in tensile strain states, respectively. (**b**), (**d**), (**f**), (**h**) and (**j**) The PPF index response of the TAS under different bending radius (20, 40, 60, 80 and 100 mm) in tensile strain states

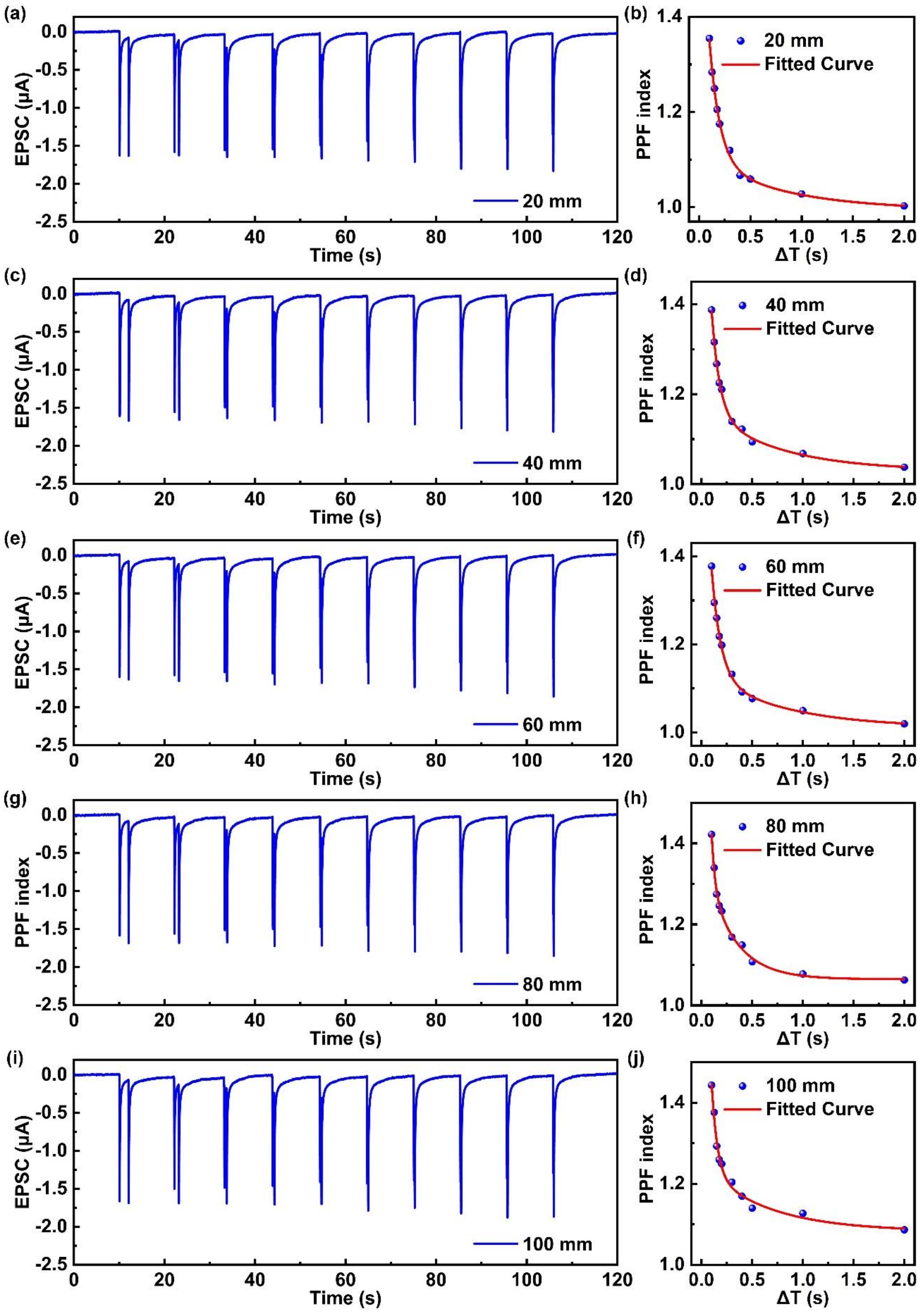

Fig. S8 The EPSC and PPF index response of the TAS under different bending radius in compressive strain states. (**a**), (**c**), (**e**), (**g**) and (**i**) The EPSC response of the TAS under different bending radius (20, 40, 60, 80 and 100 mm) in compressive strain states, respectively. (**b**), (**d**), (**f**), (**h**) and (**j**) The PPF index response of the TAS under different bending radius (20, 40, 60, 80 and 100 mm) in compressive strain states, respectively.

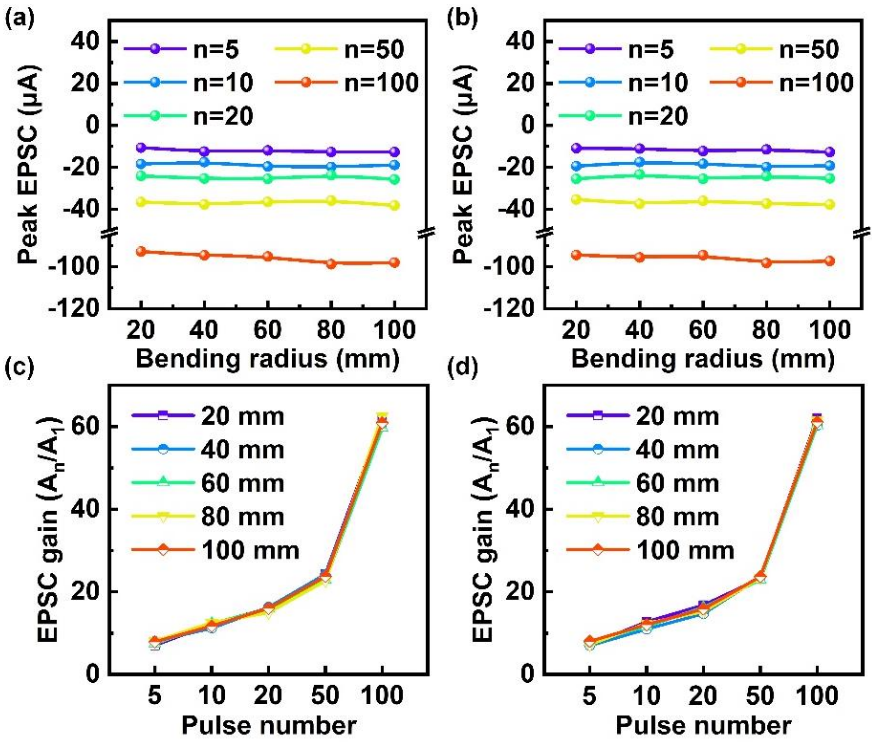

Fig. S9 The effect of different bending radius on the output characteristics of the TAS under multiple mechanical stimuli with a duration time of 0.1 s. (**a**) The effect of different bending radius in tensile strain states on the EPSC peak value of the TAS under multiple mechanical stimuli with a duration time of 0.1 s. (**b**) The effect of different bending radius in compressive strain states on the EPSC peak value of the TAS under multiple mechanical stimuli with a duration time of 0.1 s. (**c**) The effect of the different bending radius in tensile strain states on the EPSC gain of the TAS (EPSC gain is defined as A_n/A_1) under multiple continuous mechanical stimuli with a duration time of 0.1 s. (**d**) The effect of the different bending radius in compressive strain states on the EPSC gain of the TAS (EPSC gain is defined as A_n/A_1) under multiple continuous mechanical stimuli with a duration time of 0.1 s

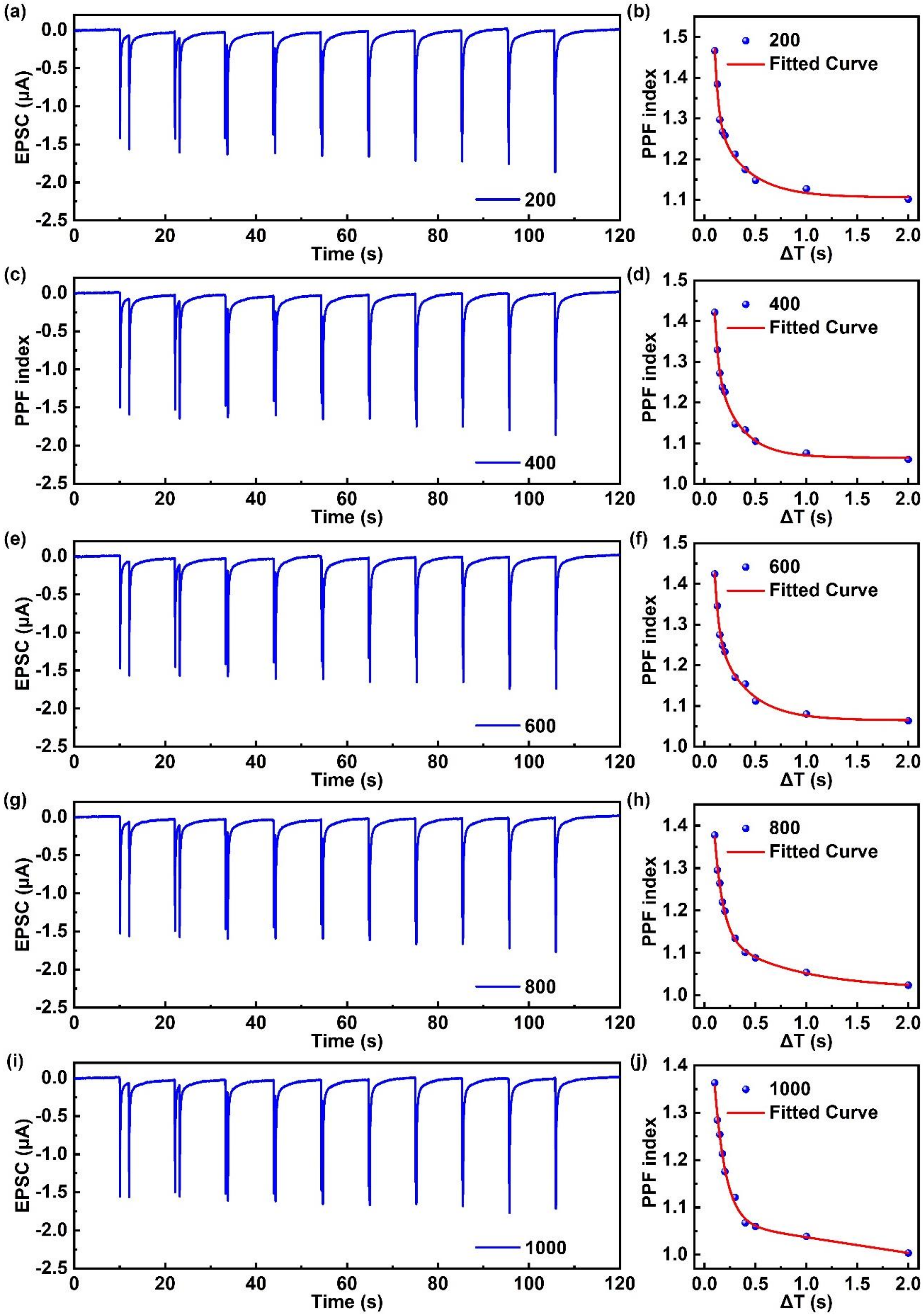

Fig. S10 EPSC and PPF index response of the TAS after tensile strain cycles with bending radius of 20 mm. (**a**), (**c**), (**e**), (**g**) and (**i**) The EPSC response of the TAS after different tensile strain cycles (200, 400, 600, 800 and 1000) with bending radius of 20 mm, respectively. (**b**), (**d**), (**f**), (**h**) and (**j**) The EPSC response of the TAS after different tensile strain cycles (200, 400, 600, 800 and 1000) with bending radius of 20 mm

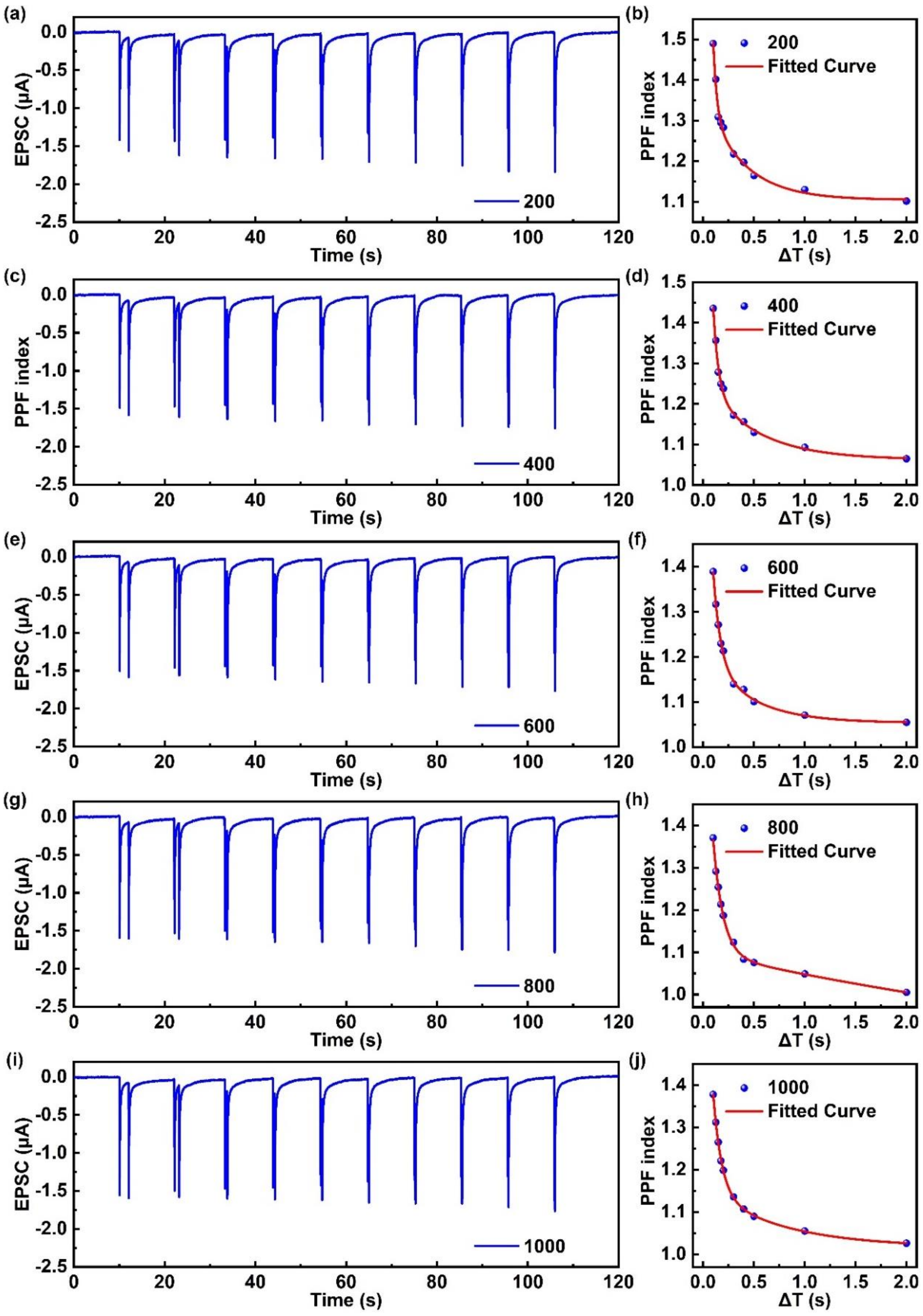

Fig. S11. The EPSC and PPF index responses of the TAS after compressive strain cycles with bending radius of 20 mm. (**a**), (**c**), (**e**), (**g**) and (**i**) The EPSC response of the TAS after different compressive strain cycles (200, 400, 600, 800 and 1000) with bending radius of 20 mm, respectively. (**b**), (**d**), (**f**), (**h**) and (**j**) The EPSC response of the TAS after different compressive strain cycles (200, 400, 600, 800 and 1000) with bending radius of 20 mm

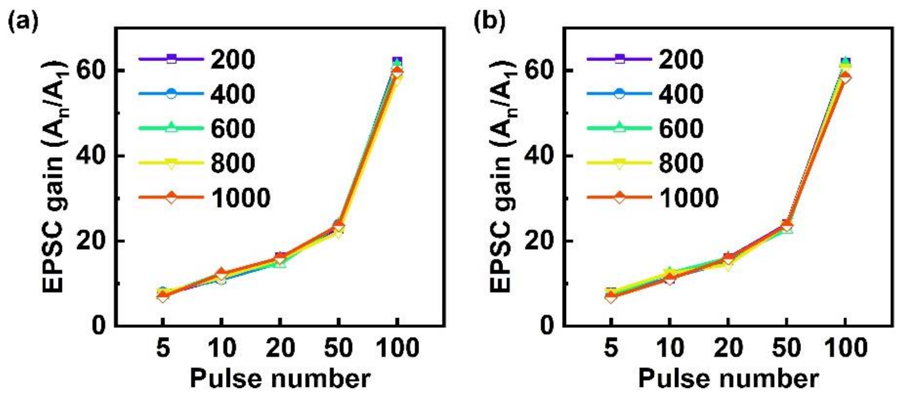

Fig. S12 The effect of the number of tensile and compressive strain cycles with a bending radius of 20 mm on the EPSC gain of the TAS (EPSC gain is defined as A_n/A_1) under multiple continuous mechanical stimuli with a duration time of 0.1 s. (**a**) The effect of the number of tensile strain cycles with a bending radius of 20 mm on the EPSC gain of the TAS under multiple continuous mechanical stimuli with a duration time of 0.1 s. (**b**) The effect of the number of compressive strain cycles with a bending radius of 20 mm on the EPSC gain of the TAS under multiple continuous mechanical stimuli with a duration time of 0.1 s

Table S1 The comparison of the reported artificial synapses in previous literature

Activation modes	Substrate	Electrodes	Active layer	Dielectric layer	Stimuli signal	EPSC Decay time	PPF A^2/A^1	SM to LTM Retention time	Refs.
External power	ITO glass	IZO	IZO	Chitosan	Electrical pulse	N/A	~1.36	8 s	[S1]
Piezoelectric	PET	Cr/Au	Graphene	PEGDA+HOMPP EMIM-TFSI	Force	3.24 s	~1.4	—	[S2]
Triboelectric	Si	Cr/Au	MoS_2	PSSH	Force	N/A	1.16	—	[S3]
	Si	Cr/Au	MoS_2	PEGDA+HOMPP EMIM-TFSI	Force	75 ms	~1.4	~10 s	[S4]
	PET	Ti/Au	P3HT-NF/PDMS	PVDF-HFP +EMIM-TFSI	Force / Vibration	6.89 s	1.56	30 s	This work

Supplementary References

[S1]J. Zhou, Y. Liu, Y. Shi, Q. Wan, Solution-processed chitosan-gated IZO-based transistors for mimicking synaptic plasticity. Ieee Electr. Device L. **35**(2), 280-282(2014). https://doi.org/10.1109/LED.2013.2295815

[S2]Y. Chen, G. Gao, J. Zhao, H. Zhang, J. Yu et al., Piezotronic graphene artificial sensory synapse. Adv. Funct. Mater. **29**(41), 1900959(2019). https://doi.org/10.1002/adfm.201900959

[S3]X. Yang, J. Han, J. Yu, Y. Chen, H. Zhang et al., Versatile triboiontronic transistorvia proton conductor. ACS Nano **14**(7), 8668-8677(2020). https://doi.org/10.1021/acsnano.0c03030

[S4]J. Yu, G. Gao, J. Huang, X. Yang, J. Han et al., Contact-electrification-activated artificial afferents at femtojoule energy. Nat. Commun. **12**(1), 1581(2021). https://doi.org/10.1038/s41467-021-21890-1
